# Cholesterol and Cardiolipin Importance in Local Anesthetics–Membrane Interactions: The Langmuir Monolayer Study

**DOI:** 10.1007/s00232-018-0055-6

**Published:** 2018-11-30

**Authors:** Justyna Mildner, Anita Wnętrzak, Patrycja Dynarowicz-Latka

**Affiliations:** Department of General Chemistry, Faculty of Chemistry, Jagiellonian University, Gronostajowa 2, 30-387 Kraków, Poland

**Keywords:** Langmuir monolayers, Membrane mimetic models, Interactions, Local anesthetics

## Abstract

Local anesthetics (LAs) are known to act on membrane level; however, the molecular mechanism of their activity is still not fully understood. One hypothesis holds that these drugs can incorporate into lipid membrane of nerve cells and in this way change conformation of channel proteins responsible for transport of sodium ions. However, the action of anesthetics is not limited to nerve cells. These drugs also affect other types of cells and organelles, causing severe side effects. In this paper, we applied Langmuir monolayers—as model of cellular membranes—and investigated interactions between selected amide-type local anesthetics (lidocaine prilocaine, mepivacaine and ropivacaine, in the form of hydrochlorides) and lipid components of natural membranes: cholesterol, POPC and cardiolipin (CL) and their mixtures (POPC/cholesterol and POPC/CL/cholesterol), which can serve as simplified models of nerve cell membranes, erythrocytes, and mitochondria. The influence of the drug was monitored by registering the surface pressure (*π*) as a function of surface area per molecule (*A*) in a monolayer in the presence of the drug in the subphase. The structure of lipid monolayers on subphases containing and devoid of the studied drugs were visualized with Brewster angle microscopy (BAM). Langmuir monolayer studies complemented with surface visualization technique reveal the expansion and fluidization of lipid monolayers, with the most pronounced effect observed for cardiolipin. In mixed systems, the effect of LAs was found to depend on cholesterol proportion. The observed fluidization of membranes by local anesthetics may negatively affect cells functioning and therefore can explain side effects of these drugs both on the cardiovascular and nervous systems.

**Electronic supplementary material:**

The online version of this article (10.1007/s00232-018-0055-6) contains supplementary material, which is available to authorized users.

## Introduction

**Scheme 1:**
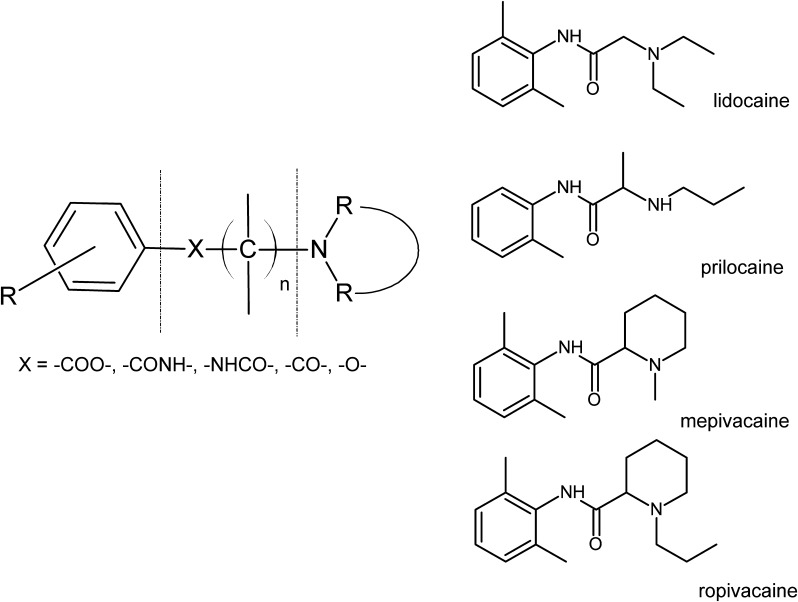
General structure of local anesthetics and chemical structure of the investigated drugs

From the second half of the nineteenth century, when the pain-relief effect of cocaine was discovered, an intensive development of research on substances with local anesthetic activity began. It was shown that there is a close relationship between the chemical structure of the molecule and its anesthetic properties. Namely, the local anesthetic (LA) has an amphipathic structure—it contains hydrophobic (lipophilic) group, composed of an aromatic ring, connected by an intermediate chain to a hydrophilic group, which is usually a secondary or tertiary amine system. In the chain linking the polar and apolar fragments, there is usually either ester or amide group; or—less often—another group (e.g., carbonyl or ether) (Scheme 1).

Therefore, based on the nature of this link, local anesthetics are classified into two main groups: aminoesters (e.g., cocaine, benzocaine) and aminoamides (e.g., lidocaine, prilocaine); both containing an ionizable amino group. These drugs are administered to the body in water-soluble form as hydrochlorides. Their pharmacological action is a result of reversible blocking of the conduction of electrical impulses across the neuronal cell membrane, resulting in change of permeability for sodium ions (Gonzalez et al. 2001). This can occur as a result of either direct bounding of drug molecules to sodium ion channels (Li et al. 1999; Butterworth and Strichartz 1990; Strichartz 1973) or by incorporation into lipid membrane, which can induce changes of its biophysical properties and, indirectly, the conformation of ion channels, causing the blocking of sodium ions transport (Strichartz and Ritchie 1987; Suwalsky et al. 2002; Smith et al. 1991). Although it has been widely accepted that some LAs (lidocaine can serve as an example) yield anesthesia through direct adsorption into the receptor, the other hypothesis, assuming changes in membrane physicochemical properties upon incorporation of LAs, cannot be ruled out. Evidence of this is the expansion and increase in fluidity of model lipid membrane that both take part in the process of anesthesia which had been demonstrated as early as in the fifties of the preceding century (Skou 1954a) and correlation between anesthetic potency and the ability of LAs to penetrate the cell membranes (Skou 1954b). Further studies (Hendrickson 1976; Seeling 1987) confirmed these findings for different LAs and lipid membranes, modelled mainly as monolayers.

Shortly after the introduction of local anesthetics in pharmacological use, a problem related to their toxicity appeared. It turned out that these drugs cause numerous side effects, including disorders of the cardiovascular and nervous systems. Since it was found that local anesthetics—due to their amphiphilic structure—can incorporate into the nerve cells membranes and interact with their lipid components, the question arose whether they can also act similarly on other types of cells. In vitro studies have shown that the action of benzocaine leads to a change in the morphology of erythrocytes, affecting the transport of oxygen in the body (Suwalsky et al. 2002). It has also been reported that local anesthetics induce a cardiotoxic effect by interacting with mitochondrial membranes of cardiomyocytes (Önyüksel et al. 2007). Any change in physicochemical properties of cell membranes may cause severe disturbances in the functioning of cells and, consequently, may be harmful to the whole organism.

Previous studies indicate that local anesthetics act on membrane level. I order to reach their binding sites on the sodium channels, they must penetrate across the lipid bilayer. Due to the presence an ionizable amino group in their structure, in aqueous medium the equilibrium is established and the uncharged form can easily cross the lipid membrane to reach its active site. Therefore, to get insight into their anesthetic activity as well as side effects it is of great importance to perform systematic investigations of the interactions between the drugs and model membranes mimicking various cells, namely nerve cells, erythrocytes and mitochondria. However, the lipid composition of these systems is complex, and therefore it is useful to apply one of the membrane models. Different models mimicking cell membrane can be chosen (reviewed in Peetla et al. 2009; Chan and Boxer 2007); however, most popular are liposomes/vesicles (Kell 1981) and Langmuir monolayers (Nobre et al. 2015; Stefaniu et al. 2014). Langmuir monolayers are advantageous compared to other model membrane systems because they allow easy control of parameters such as molecular packing, physical state, lateral pressure, and composition. In addition, with the Langmuir technique, it is possible to mimic similar conditions as in the natural membrane. It was found that the pressure in biological membranes corresponds to the surface pressure of 30–35 mN/m in the Langmuir experiment (Marsh 2006).

With all the above in mind, and taking into consideration that LAs have mainly been investigated with liposomes (Mizogami et al. 2002; Tsuchiya and Mizogami 2008; Tsuchiya et al. 2010), in our study we have applied the monolayer technique (Gaines 1966) to mimic biomembrane. First, we applied one-component systems formed by membrane lipids, and—in the next step—we extended the investigations into more complex, multicomponent systems that account for a particular model membrane. Since erythrocyte and nerve cell membranes can be modeled as POPC/Chol system of different cholesterol contents while mitochondrion as POPC/CL/Chol (Fewster et al. 1976; Jamieson and Robinson 1977), in the first step of our investigations we have performed experiments with LAs interacting with particular membrane lipids (POPC, CL, cholesterol). As regards LAs, since former studies mainly involved ester-type drugs, for our research four amide-type drugs, lidocaine, prilocaine, mepivacaine, and ropivacaine (Scheme 1), differing basically in the structure of the amine fragment, have been chosen.

Our study was based on penetration experiments of the chosen LA hydrochlorides (LiC, PriC, MC, and RC, respectively), dissolved in aqueous subphase, into monolayers formed by the above-mentioned membrane lipids and their mixtures. Thermodynamic parameters of interaction and visualization of films structure with Brewster angle microscopy (BAM) were applied to characterize the investigated systems.

## Experimental

### Materials

The investigated drugs, lidocaine hydrochloride, prilocaine hydrochloride, mepivacaine hydrochloride, and ropivacaine hydrochloride (in the form of powders, purity ≥ 98%), were purchased from AK Scientific Inc., and CHEMOS GmbH. The following membrane lipids were used: 1-palmitoyl-2-oleoyl-sn-glycero-3-phosphocholine (POPC), cholesterol (Chol) and bovine brain cardiolipin (CL); all were synthetic products of high purity (≥ 99%) purchased from Sigma (Chol) and Avanti Polar Lipids, Inc. (POPC, CL). Spreading solutions of lipids were prepared in chloroform/methanol 9:1 (v/v) mixture, (HPLC grade, ≥ 99.9%, Aldrich).

### Methods

#### Langmuir Monolayer Technique

The aliquots of the studied lipids were dropped onto aqueous subphase with the Hamilton microsyringe, precise to 10 µL. To investigate the influence of local anesthetics on model lipid membrane, the monolayers were spread on pure water and aqueous solutions of particular drugs of the concentration 1 mM, which corresponds to their bloodstream concentration. After spreading, the lipid monolayers were left to equilibrate for 5 min (pure water) and 15 min (drug solutions) before the compression was started. In all the experiments, monolayers were compressed with barrier speed of 20 cm^2^/min. *π*–*A* isotherms were recorded with a NIMA (U.K.) Langmuir trough (total area = 300 cm^2^) equipped in one movable barrier, placed on an anti-vibration table. Surface pressure was measured with the accuracy of ± 0.1 mN/m using a Wilhelmy plate made of filter paper (ashless Whatman Chr1) connected to an electrobalance. The subphase temperature (20 °C) was controlled thermostatically to within 0.1 °C by a circulating water system. All experiments were repeated at least twice to ensure good reproducibility of the isotherms (the error for the area per molecule and surface pressure did not exceed 0.1 Å^2^/molecule and 0.1 mN/m, respectively). In all the experiments, Milli-Q water (resistivity of 18.2 MΩ cm; pH 6) was used.

*Brewster angle microscopy (BAM)* experiments were performed with UltraBAM instrument (Accurion GmbH, Goettingen, Germany) equipped with a 50 mW laser emitting *p*-polarized light at a wavelength of 658 nm, a × 10 magnification objective, polarizer, analyzer and a CCD camera. The spatial resolution of BAM was 2 µm.

## Results and Discussion

### Local Anesthetics in One-Component Lipid Monolayers

**Fig. 1:**
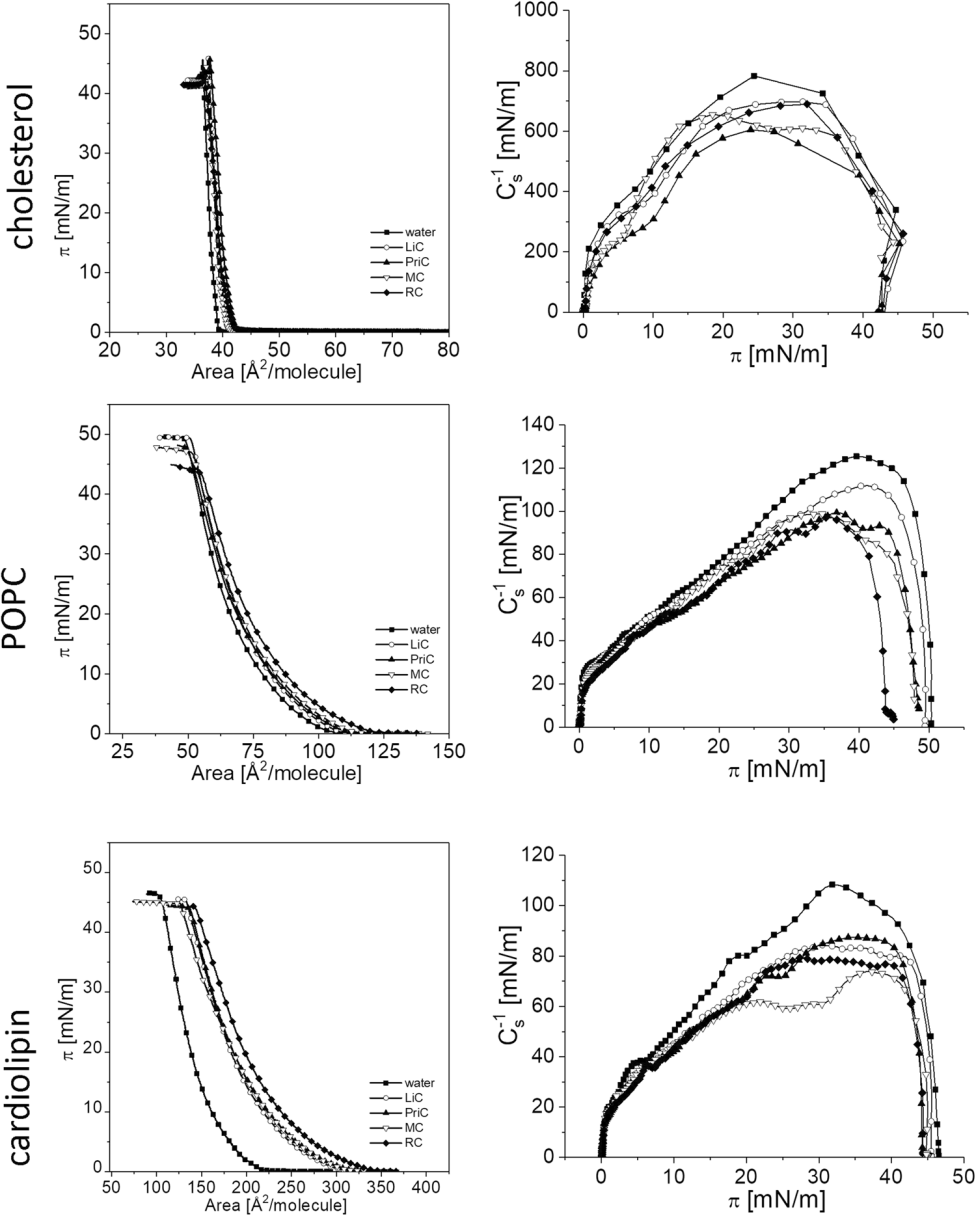
Left panel: *π*–*A* isotherms recorded for lipid monolayers of cholesterol, POPC and cardiolipin spread on water and on aqueous solution of the investigated LA hydrochlorides. Right panel: compressibility modulus ($C_{{\text{S}}}^{{ - 1}}$) versus surface pressure (*π*) dependencies

The surface pressure-area isotherms recorded for lipid monolayers on water and aqueous solution of lidocaine, prilocaine, mepivacaine, and ropivacaine hydrochlorides are presented in Fig. 1.

The isotherms of the studied lipids on water subphase are in good agreement with those published elsewhere (Dynarowicz-Latka et al. 2015; Phan and Shin 2015). The exchange of the water subphase for aqueous solution of all the studied LA hydrochlorides shifts the isotherms towards larger areas in each case, being indication of a possible penetration of drug molecules into lipid monolayer. It is important to mention that in preliminary experiments all the investigated LAs were found to be incapable to adsorb at the air/water interface without the presence of lipid monolayer.

**Fig. 2:**
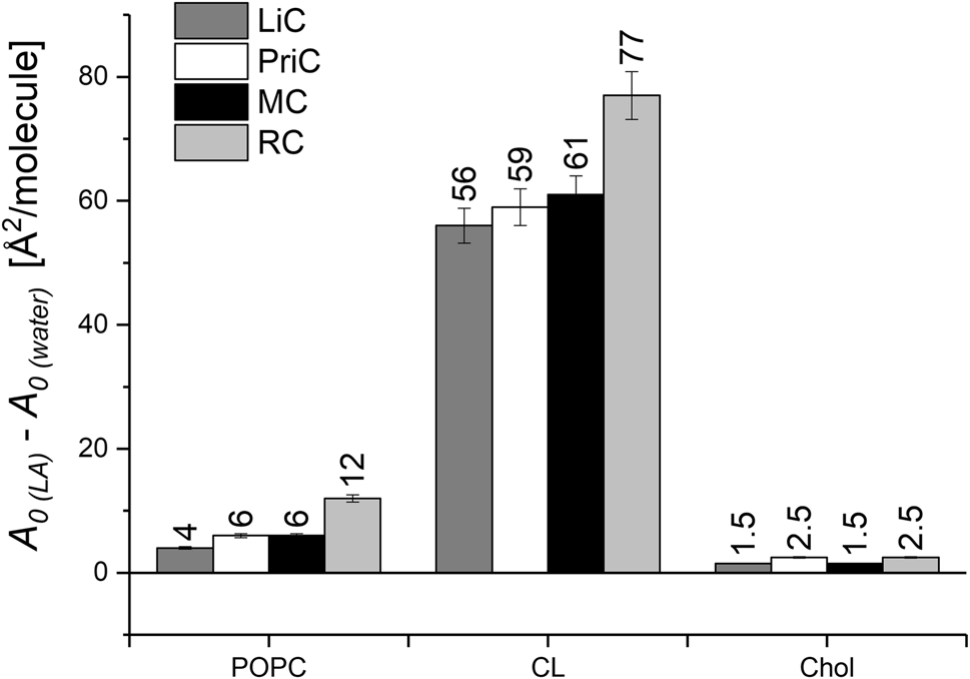
Expansion of lipid monolayers from POPC, CL, and Chol on LAs-containing subphases. *A*_0_ denotes the *lift-off* area of the surface pressure

For monolayers formed by lipids of different structures (POPC, CL, cholesterol), the most pronounced increase in *lift-off* area (*A*_0_, defined as the molecular area at the surface pressure rise) is observed for cardiolipin, while for cholesterol the effect is negligible (Fig. 2). This indicates that an important role in the expansion of the membrane lipid monolayer by LAs is played by electrostatic interactions. A negatively charged phosphate group of phospholipids interacts with positively charged drug ions, thus helping to incorporate LAs into the monolayer structure. In the case of cardiolipin, a double negative charge attracts two drug ions to one cardiolipin molecule, which increases the expansion effect. For POPC, monolayer expansion is not so significant (10–20 times lower than for cardiolipin).

The observed expansion is associated with films fluidization, which is proved by lower values of compressibility modulus $\left( {C_{{{\text{s}}~}}^{{ - 1}}} \right)$, also called “surface elasticity,” which have been calculated from the datapoints of the experimental *π*–*A* isotherms, using the following equation:

1 $$C_{{\text{s}}}^{{ - 1}}= - A{\left( {\frac{{{\text{d}}\pi }}{{{\text{d}}A}}} \right)_T}$$

where *A* is the area per molecule at a given surface pressure. This parameter is useful to identify the physical state of a monolayer (Davies and Rideal 1961). From the comparison of $C_{{{\text{s}}~}}^{{ - 1}}$ values for the investigated lipid monolayers spread on water (Fig. 1, right panel), it is evident that the physical state of the studied membrane lipids is different and most condensed monolayer can be achieved by cholesterol, while the most expanded is formed by cardiolipin.

**Fig. 3:**
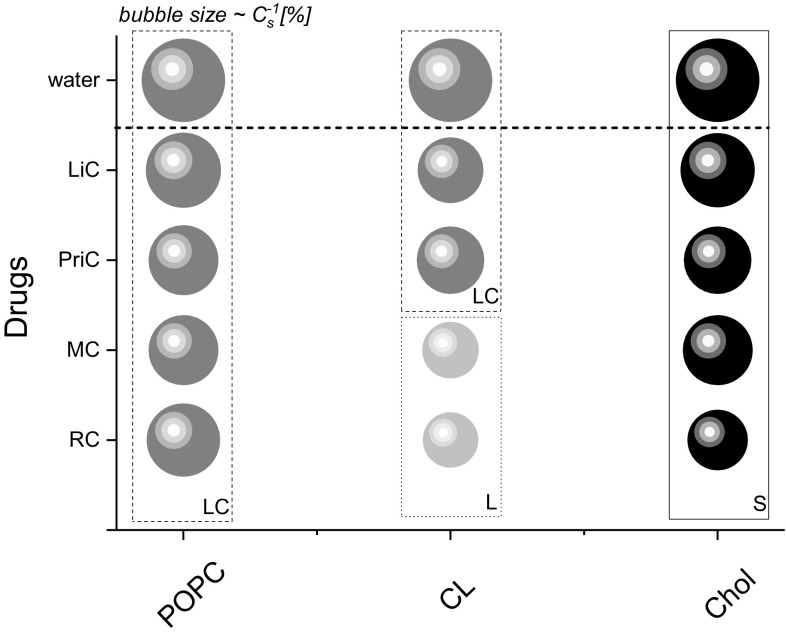
The effect of LA on physical state of monolayer formed by different lipids; *L* liquid, *LC* liquid condensed, *S* solid state

It is interesting to point out that in the presence of LAs in the subphase, profiles of the $C_{{{\text{s}}~}}^{{ - 1}}$ versus *π* curves do not change substantially, implying that LA–lipid interactions are not associated with any substantial change in lipid molecules arrangement. However, the decrease in surface elasticity is clearly visible, meaning that in the presence of LAs, lipid monolayers undergo fluidization. The observed fluidization with LiC, PriC, MC, and RC in the subphase is consistent with previous results carried out for tetracaine on DMPC monolayer (Lee et al. 2002), lidocaine and tetracaine on DPPC and DMPA monolayers (Butterworth and Strichartz 1990) and lidocaine on phosphatidylcholine and phosphatidylethanolamine monolayers (Strichartz 1973). Figure 3 illustrates changes of physical states of lipid monolayers upon introduction of the studied LAs. The diameter of bubbles is proportional to the compression modulus value [%], where 100% corresponds to the value for lipid films formed on water; the physical state of monolayers was determined according to compression modulus values.

From Fig. 3, it is clearly seen that the fluidization of lipid monolayers occurs in all the cases; however, these changes occur within the same physical state of monolayer, except for MC and RC on cardiolipin monolayer, where the fluidizing effect is the strongest.

Another conclusion that can be drawn upon analyzing the isotherms recorded on water and LAs aqueous solutions is that the presence of the drug decreases the stability of each studied lipid monolayer as evidenced by its lower collapse pressure.

The observed from the *π*/*A* curves expansion and fluidization of lipid monolayers in the presence of LAs has been found to be of comparable extent for all the studied drugs and no important relationship can be found between the extent of the observed phenomena and chemical structure of the studied drugs. This is quite understandable taking into consideration electrostatic nature of the drug–membrane lipid interactions.

The fluidizing effect has been additionally proved using microscopic surface visualization technique—Brewster angle microscopy. General observation is that lipid monolayers images become richer in darker areas upon incorporation of LAs molecules, which is especially visible in gaseous state (*π* ~ 0) at large molecule areas, confirming films fluidization (Supplementary Material 1, Figs. S1.1–S1.3). Since the effect has been found to be similar for all the studied LAs, the results are presented for one selected drugs (PriC).

### Local Anesthetics in Two-Component (POPC/Chol) Monolayers

After looking at the behavior of LAs on monolayers from particular membrane lipids, in the next step of investigations we have advanced the lipid system for two-components mixtures of POPC/Chol, which can account for a simplified model of biomembrane. Since the influence of all the investigated drugs is very similar, the results are exemplified for prilocaine hydrochloride.

**Fig. 4:**
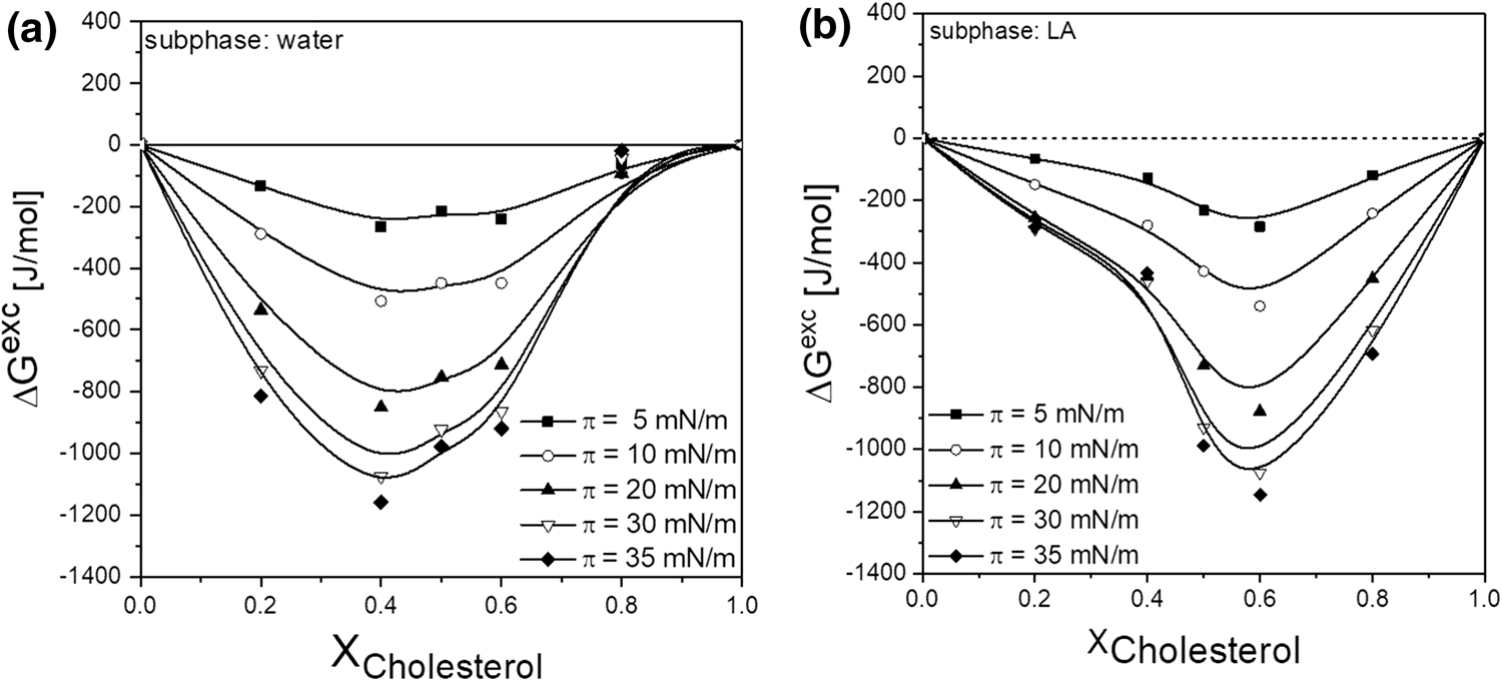
The excess free enthalpy of mixing values (∆*G*^exc^) versus film composition for POPC/chol system on water (**a**) and drug (prilocaine, PriC) solution (**b**)

Figure 4 shows the thermodynamic parameter of interaction: excess free enthalpy (Δ*G*^exc^) versus film compositions for POPC/chol system on water (a) and drug (prilocaine, PriC) solution (b), while the isotherms and compressibility moduli dependencies are shown in Supplementary Material 2.

POPC/Chol system on water subphase has already been investigated and the results are in a good agreement with those already published elsewhere (Dynarowicz-Latka et al. 2015; Jurak 2013). Compressibility moduli curves confirm well-known condensing effect of cholesterol on phospholipids. Comparing the curves presented in Supplementary Material 2, Fig. S2.1, it is evident that with the drug surface elasticity of lipids decreases.

In order to get insight into drugs–model membrane interactions, excess free enthalpy of mixing, Δ*G*^exc^ (defined as $\int_{0}^{\pi } {{A^{{\text{exc}}}}d\pi }$ wherein *A*^exc^ is the difference between the mean molecular area *A*_12_ observed at a given *π* value for a particular mole ratio of the components and the ideal mean molecular area *A*_12_^id^ which is defined as the weighted average of the mean molecular areas (*A*_1_ and *A*_2_) observed for the one component monolayers (*A*_12_^id^ = *A*_1_*X*_1_ + *A*_2_(1 − *X*_1_), where *X*_1_ is the mole ratio of component 1 in the binary film). The Δ*G*^exc^ values calculated at the selected surface pressures for POPC/cholesterol mixtures on water and PriC solution are shown in Fig. 4a and b, respectively. The course of Δ*G*^exc^ versus film composition plots prove the nonideal behavior of the studied system. Negative values of Δ*G*^exc^ in the whole range of monolayer compositions indicate that the insertion of cholesterol into the phospholipid monolayer results in attractive interactions and leads to the formation of stable films. The monolayer containing *X*_Chol_ = 0.4 is of the highest thermodynamic stability (most negative Δ*G*^exc^ values). Such a minimum on the Δ*G*^exc^ = *f*(*X*) dependencies is attributed by the majority of authors to the formation of stable complexes between film molecules. Particularly for phosphatidylcholine–cholesterol mixtures, the formation of complexes has been undoubtedly confirmed (Petelska and Figaszewski 1998; Brzozowska and Figaszewski 2002a, Brzozowska and Figaszewski 2002b).

Comparison of the plots of Δ*G*^exc^ = *f*(*X*) (Fig. 4a vs. b) indicates that the magnitude of interactions is not significantly influenced by the drug (~ 1100 J/mole at the minimum both on water and LA solution); however, the minimum shifts from *X*_Chol_ = 0.4 on water to 0.6 on solution of LA as represented by PC. This means that the stoichiometry of PC–Chol complexes changes to cholesterol-richer complexes with the presence of LA.

**Fig. 5:**
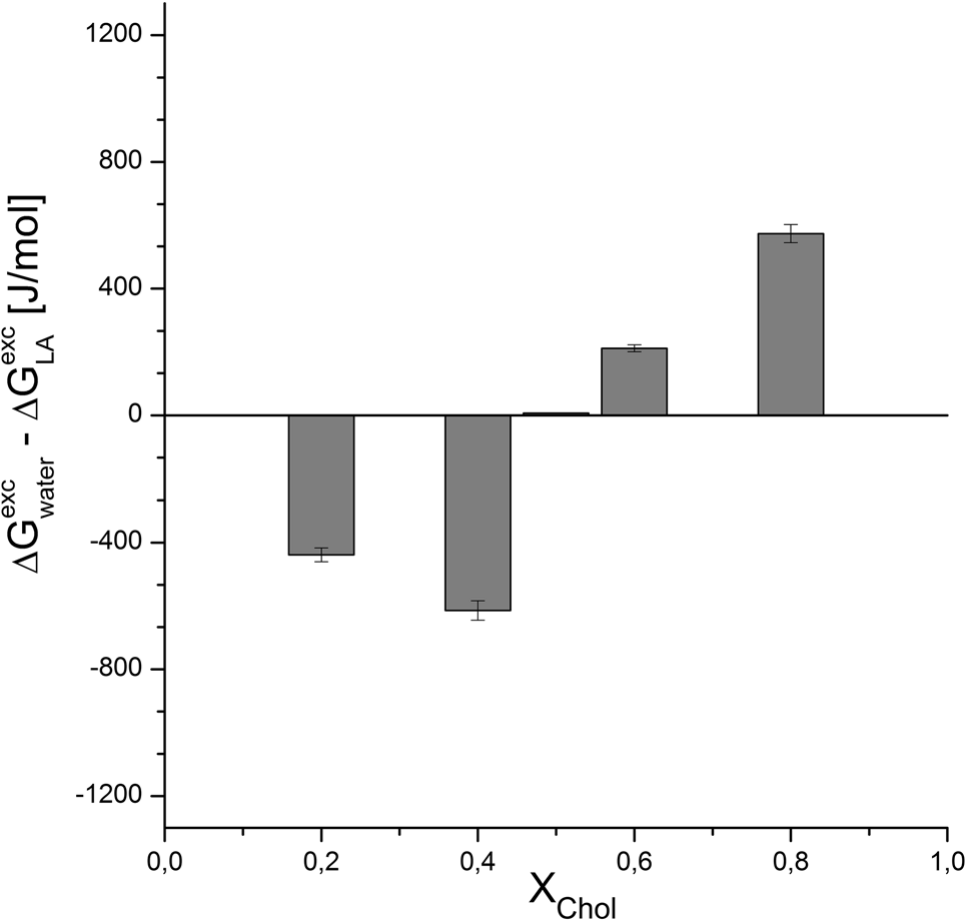
Influence of the presence of local anesthetics (represented by PriC) on ∆*G*^exc^ changes for mixed POPC/cholesterol monolayers at *π* = 30 mN/m

The excess free enthalpy values for POPC/Chol monolayers formed on the subphase containing LA and on water are presented in Fig. 5. The values have been taken at the surface pressure of 30 mN/m, which corresponds to the pressure in living systems (Marsh 2006). Negative values indicate the increase of Δ*G*^exc^ in the presence of the drug in the subphase in relation to water. This means that attractive interactions between the phospholipid and cholesterol molecules in the mixed monolayer are weakened after introducing LA into the subphase. Such a destabilizing effect of all the studied drugs on lipid monolayers is observed when cholesterol content is low (*X*_chol_ = 0.2; 0.4). For higher molar values of cholesterol (*X*_chol_ = 0.5; 0.6; 0.8), each of the investigated drugs—LiC, PriC, MC, and RC—exerted a stabilizing effect on the lipid film, as exemplified by prilocaine (Fig. 5).

The obtained results show that the amount of cholesterol is of fundamental importance as the activity of local anesthetics on the cellular membrane is concerned. It has been established that the cholesterol content in plasma membranes of various cells/tissues is different (Jamieson and Robinson 1997; Lipowsky and Sackmann 1995). For example, neurons contain low amount of cholesterol (PC/Chol = 5:1). Therefore, it can be expected that the effect of LAs on nerve cells will cause membrane destabilization contrary to other kinds of cells/organelles that are richer in cholesterol (e.g., erythrocytes, which membrane composition is reported as PC/Chol = 0.9).

### Local Anesthetics in Three-Component (POPC/Chol/CL) Monolayers

In the next step of our investigations, we have increased the complexity of the simplest membrane system by introducing cardiolipin (CL), which is an important component of mitochondrion membrane (Jamienson and Robinson 1997; Lipowsky and Sackmann 1995).

The proportion of POPC/CL reflected the composition in natural mitochondria membranes (1:0.35) (Jamieson and Robinson 1977). Although the amount of cholesterol in these membranes corresponds to *X*_Chol_ = 0.2, we have investigated also its excess on the properties of a model membrane as well as the effect of LAs on cholesterol-rich membranes.

It is evident that the presence of LAs in the subphase fluidizes the monolayer in the whole range of molar fractions studied, but for low cholesterol content (corresponding to model mitochondrion membrane; *X*_Chol_ = 0.2) this effect is not significant (Supplementary Material 2, Fig. S.2.2).

**Fig. 6:**
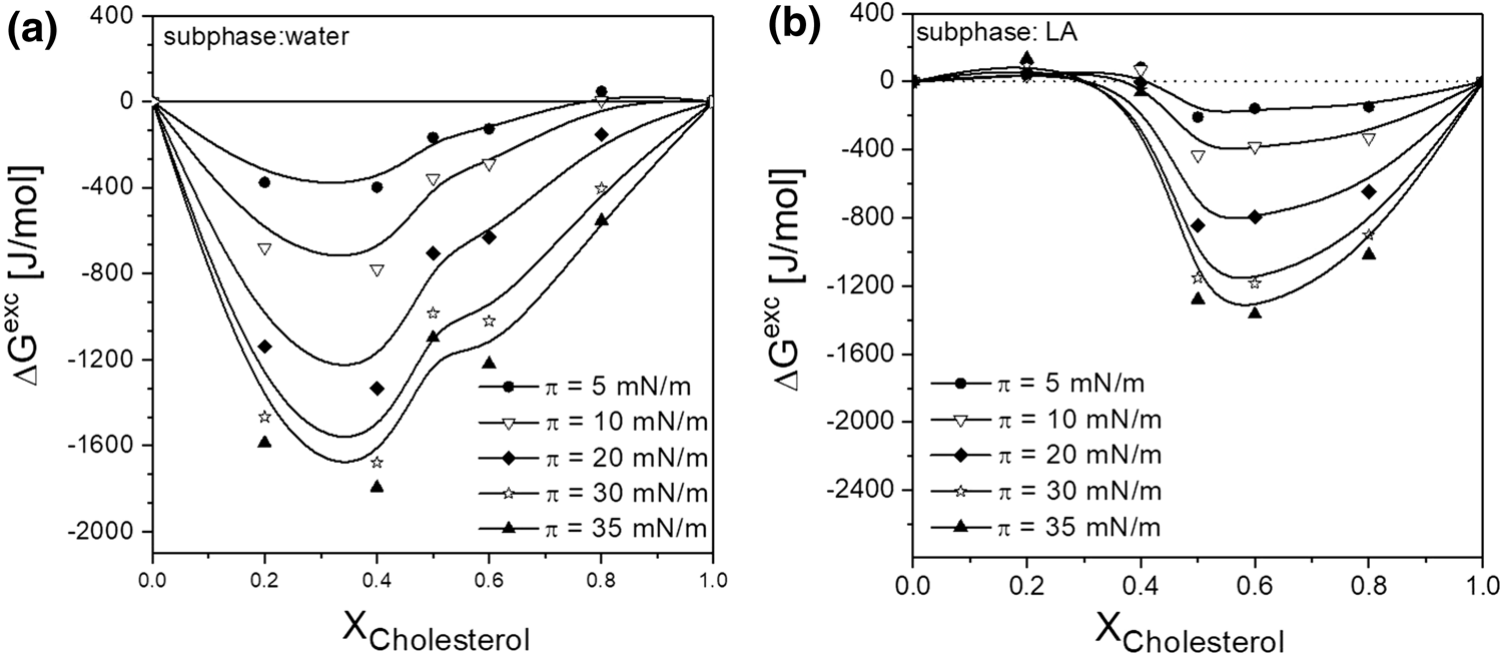
The excess free enthalpy of mixing values (∆*G*^exc^) versus film composition for POPC/CL/chol system on water (**a**) and PriC solution (**b**)

Considering the values of the excess free enthalpy of mixing (Fig. 6), it can be noticed that for low molar fractions of cholesterol (0.2, 0.4) weakening of attractive interactions between lipid molecules in mixed monolayer is observed, while for higher *X*_Chol_ the strength of attractive interactions between monolayer lipids increases and the monolayer stability is increased. It is this evident that for the model membrane of mitochondrion (*X*_Chol_ = 0.2), the destabilizing effect of LAs occurs.

**Fig. 7:**
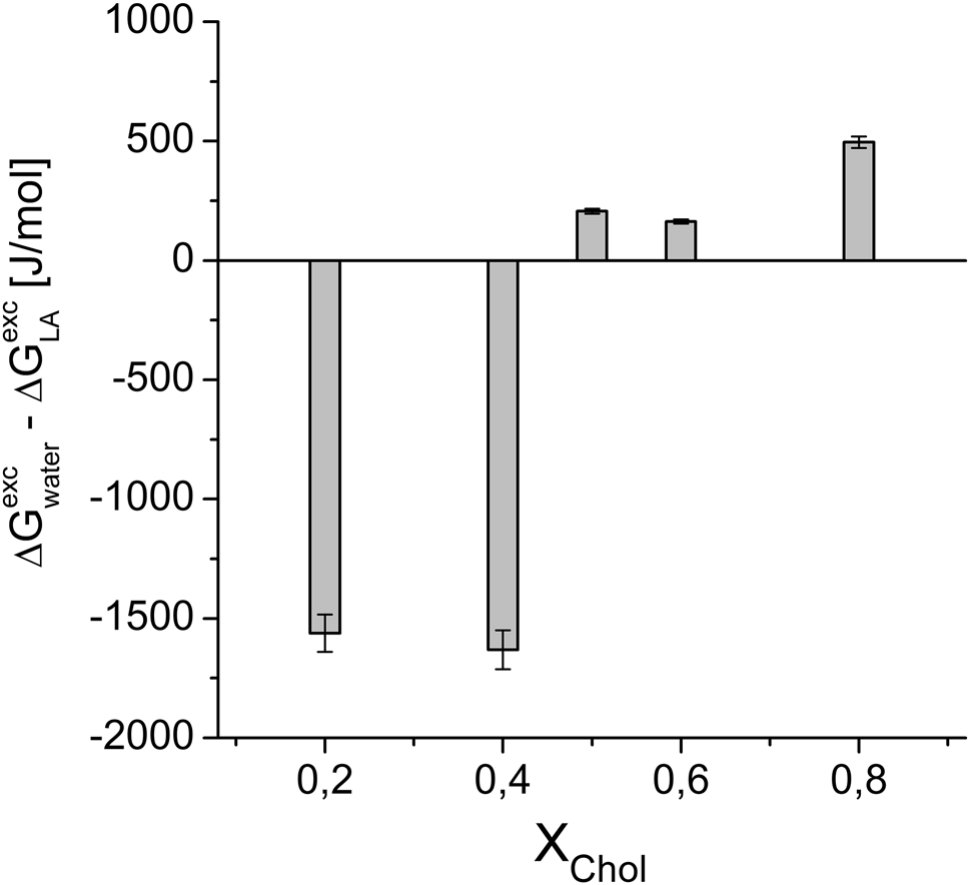
Influence of the presence of local anesthetics (represented by PriC) on ∆*G*^exc^ changes for mixed POPC/CL/cholesterol monolayers at *π* = 30 mN/m

Comparing the diagrams in Figs. 5 and 7, showing changes in Δ*G*^exc^ values for POPC/Chol and POPC/CL/Chol systems after introducing LA into the subphase, some similarities can be noticed. For both systems—at low *X*_Chol_ values—there is a destabilizing effect of the studied drugs on model membrane, while stabilization appears for cholesterol-rich membranes. Since the observed changes are greater for ternary versus binary lipid monolayers, it implies that the magnitude of changes in the Δ*G*^exc^ values must correspond to cardiolipin. This effect may explain the toxic effect of LAs on myocyte mitochondria, which membrane contains a significant amount of cardiolipin (ca. 20 mol%). Interestingly, a series of LAs cardiac toxicity (bupivacaine > > ropivacaine > lidocaine ≥ prilocaine) (Heavner 2002) follows the extent of expansion and fluidization for CL monolayer obtained in this study (Figs. 2, 4).

## Conclusions

Our results indicate that local anesthetics have significant impact on membrane lipids and can influence both nerve cells, erythrocytes and mitochondria. It is well known that optimal membrane fluidity is of great importance for normal cells functioning and any deviation may cause their dysfunction and can explain the observed side effects of LAs both on the cardiovascular and nervous systems.

## Electronic supplementary material

Below is the link to the electronic supplementary material.

Supplementary material 1 (DOCX 2177 KB)

Supplementary material 2 (DOCX 469 KB)
