# Supplementary material for: Cholesterol and Cardiolipin Importance in Local Anesthetics–Membrane Interactions: The Langmuir Monolayer Study
Source: J Membr Biol. 2018 Nov 30;252(1):31–9. doi: 10.1007/s00232-018-0055-6 (PMC6514108; doi:10.1007/s00232-018-0055-6)
Supplement: Supplementary file 1 — Supplementary material 1 (DOCX 2177 KB) [file 232_2018_55_MOESM1_ESM.docx]

**Supplementary Material 1**

The Journal of Membrane Biology

**Cholesterol and cardiolipin importance in local anesthetics-membrane interactions – the Langmuir monolayer study**

Justyna Mildner, Anita Wnętrzak, Patrycja Dynarowicz-Latka^*)^

*Corresponding author: [ucdynaro@cyf-kr.edu.pl](mailto:ucdynaro@cyf-kr.edu.pl)

| 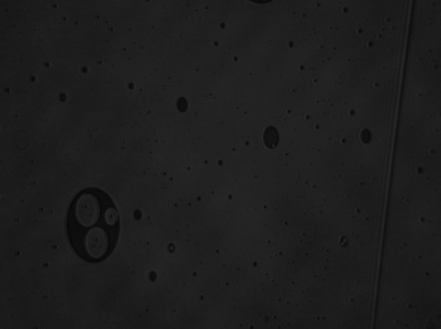  a  0 mN/m | 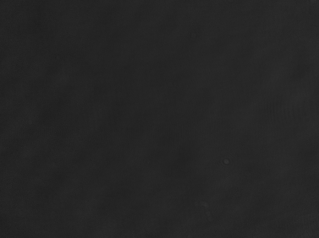  b  18-43 mN/m | 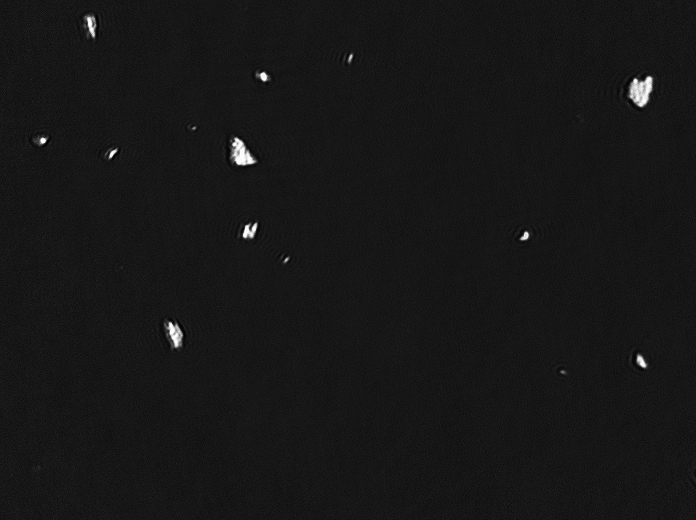  c  44 mN/m |
| --- | --- | --- |
| 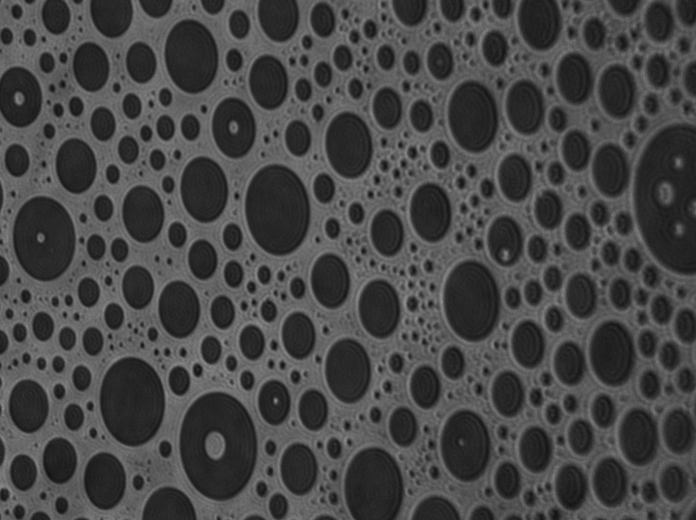  0 mN/m  db |   18-44 mN/m  e |   45 mN/m  f |
|  |  |  |

**Fig. S1.1.** BAM images for cholesterol monolayer spread on water (a, b, c) and on drug (PriC) solution (d, e, f).

| 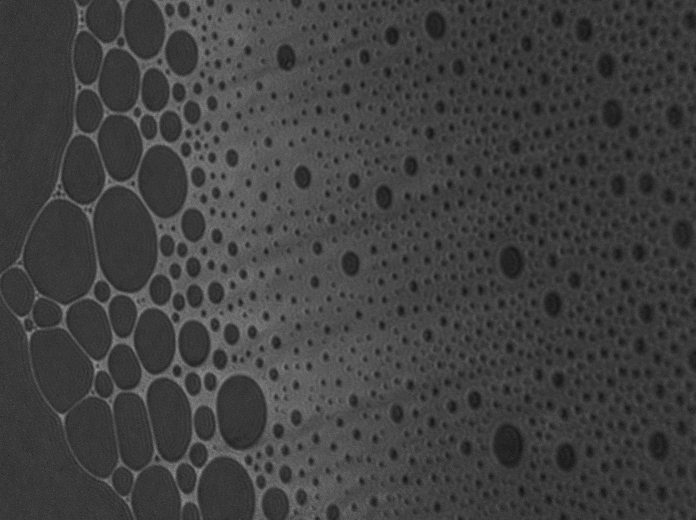  a  0 mN/m |   16 mN/m  b | 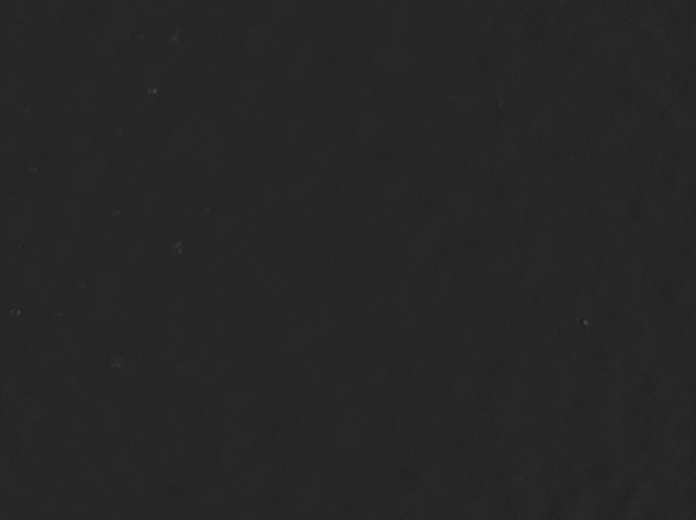  47 mN/m  c |
| --- | --- | --- |
|   db  0 mN/m |   14 mN/m  e |   f  43 mN/m |
| 0 mN/m | 16 mN/m | 46 mN/m |

**Fig. S1.2.**  BAM images for POPC monolayer spread on water (a,b,c) and on drug (PriC) solution (d,e,f).

|   0 mN/m  a b c | 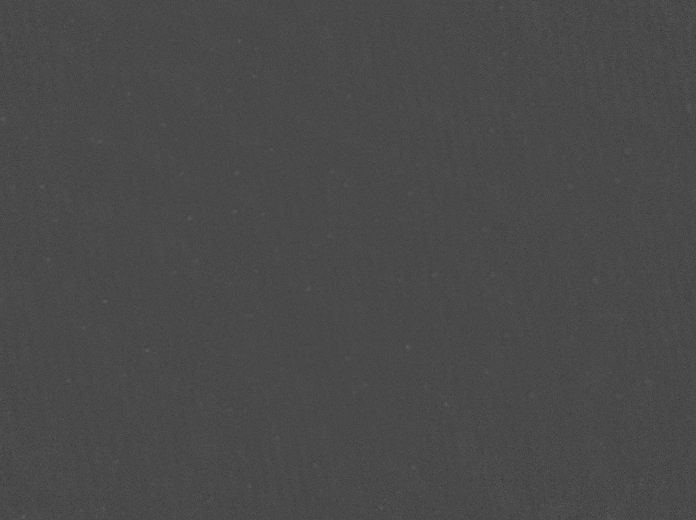  10 mN/m | 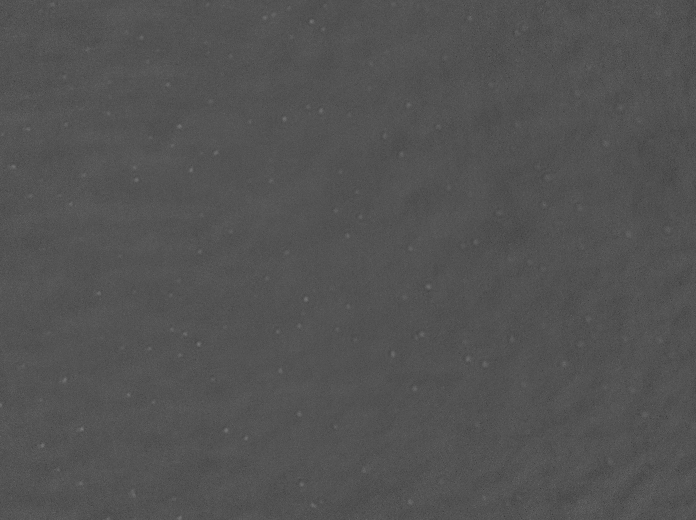  37 mN/m |
| --- | --- | --- |
| 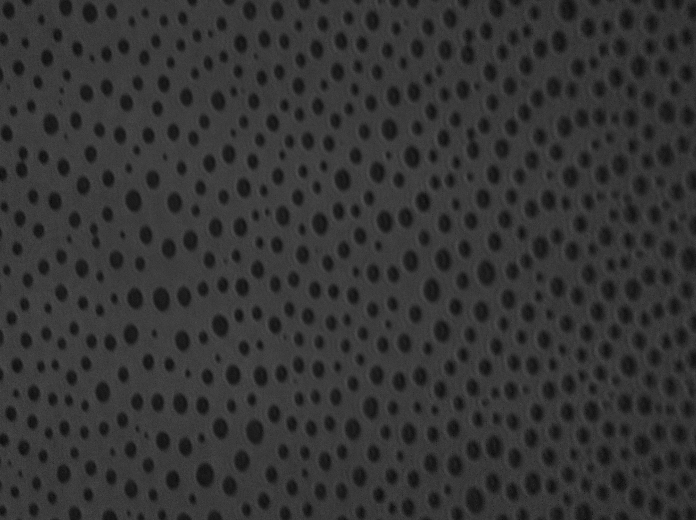  0 mN/m  d e f | 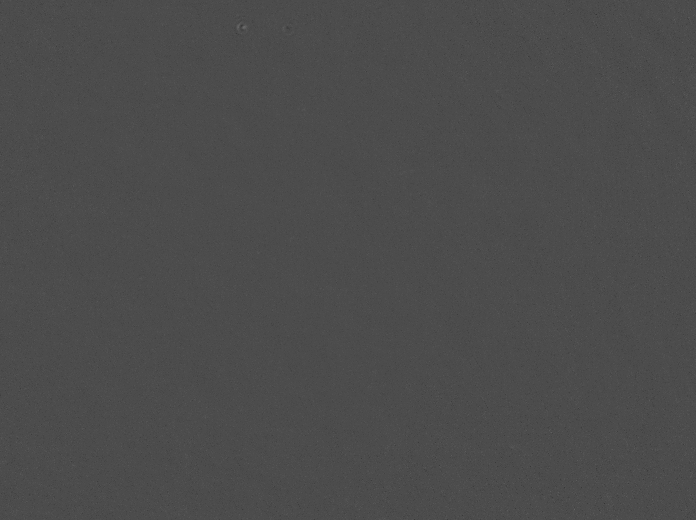  10 mN/m | 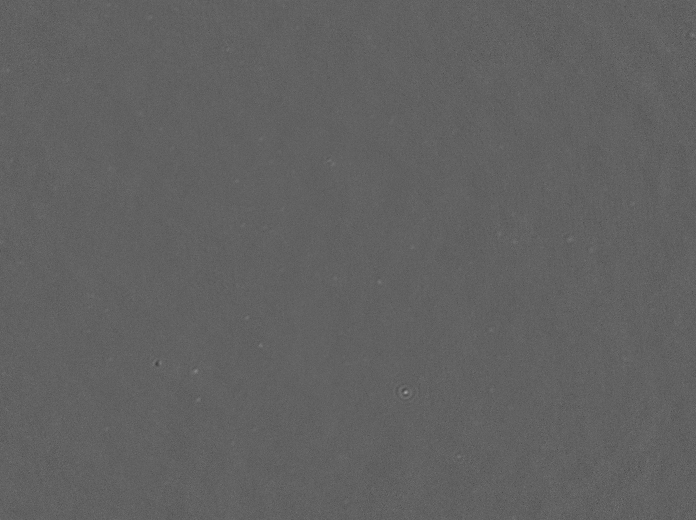  41 mN/m |
|  | 10 mN/m | 43 mN/m |

**Fig. S1.3.**. BAM images for CL monolayer spread on water (a,b,c) and on drug (PriC) solution (d,e,f).
