# Supplementary material for: Cholesterol and Cardiolipin Importance in Local Anesthetics–Membrane Interactions: The Langmuir Monolayer Study
Source: J Membr Biol. 2018 Nov 30;252(1):31–9. doi: 10.1007/s00232-018-0055-6 (PMC6514108; doi:10.1007/s00232-018-0055-6)
Supplement: Supplementary file 2 — Supplementary material 2 (DOCX 469 KB) [file 232_2018_55_MOESM2_ESM.docx]

**Supplementary Material 2**

The Journal of Membrane Biology

**Cholesterol and cardiolipin importance in local anesthetics-membrane interactions – the Langmuir monolayer study**

Justyna Mildner, Anita Wnętrzak, Patrycja Dynarowicz-Latka^*)^

*Corresponding author: [ucdynaro@cyf-kr.edu.pl](mailto:ucdynaro@cyf-kr.edu.pl)


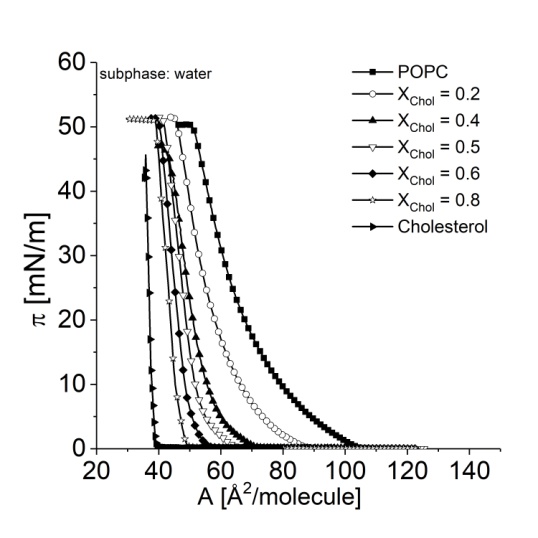

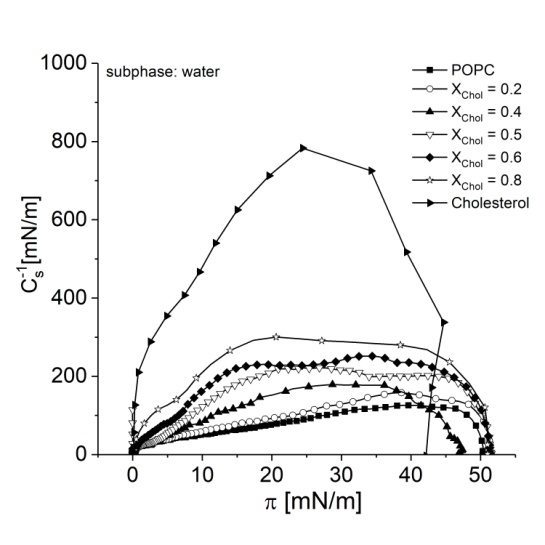


d)

b)

a)


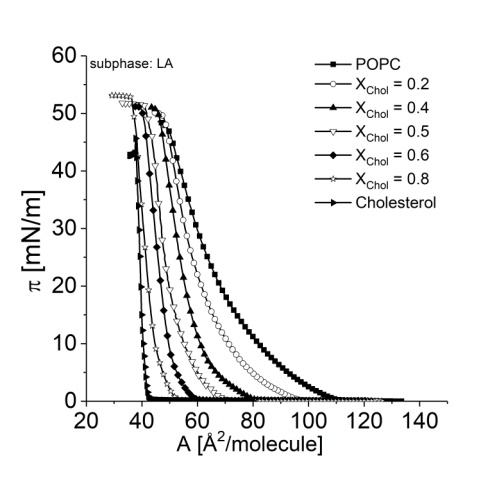

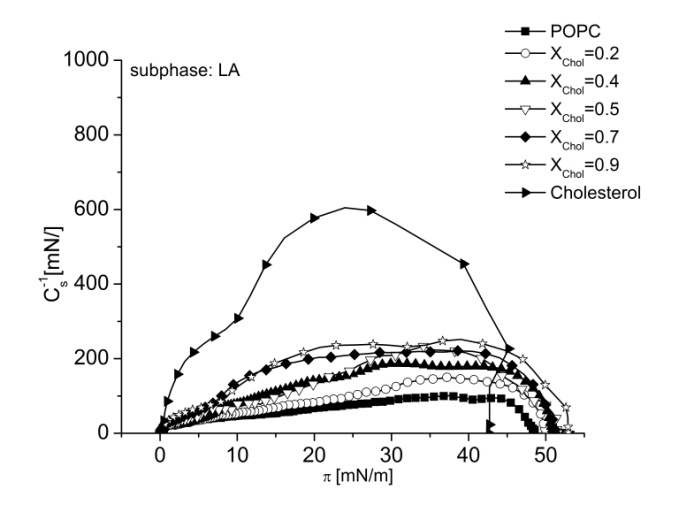


c)

**Fig. S.2.1.** π-A isotherms, compressibility modulus ($C_{s}^{-1}$) vs surface pressure (π) dependencies for POPC/chol system on water (a, b) and on drug (PriC) solution (c, d).

**
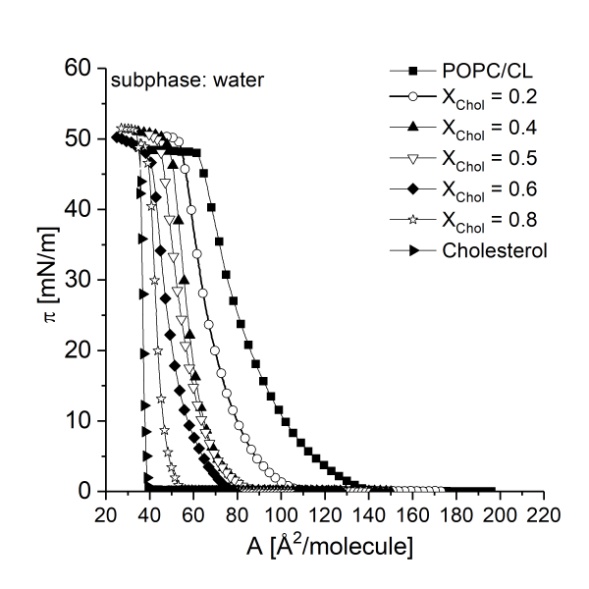

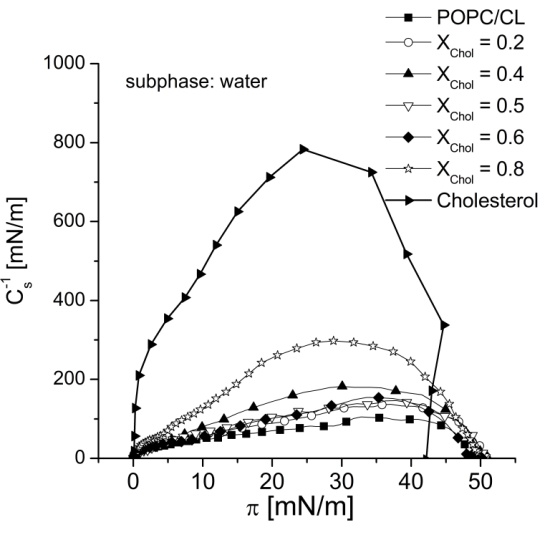
**
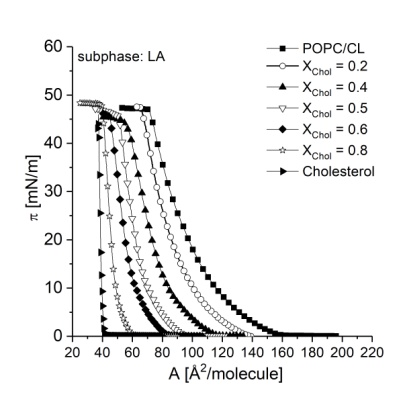

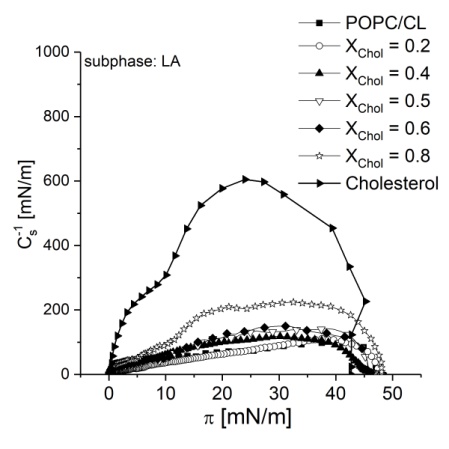


d)

b)

c)

a)

**Fig. S.2.2.** π-A isotherms, compressibility modulus ($C_{s}^{-1}$) vs surface pressure (π) dependencies for POPC/CL/chol system on water (a, b) and on drug (PriC) solution (c, d).
